# Integrated mobile genetic elements in Thaumarchaeota

**DOI:** 10.1111/1462-2920.14564

**Published:** 2019-03-18

**Authors:** Mart Krupovic, Kira S. Makarova, Yuri I. Wolf, Sofia Medvedeva, David Prangishvili, Patrick Forterre, Eugene V. Koonin

**Affiliations:** ^1^ Institut Pasteur Unité Biologie Moléculaire du Gène chez les Extrêmophiles, 75015 Paris France; ^2^ National Center for Biotechnology Information National Library of Medicine Bethesda MD 20894 USA; ^3^ Center of Life Sciences Skolkovo Institute of Science and Technology Skolkovo Russia; ^4^ Sorbonne Université Collège doctoral, 75005 Paris France; ^5^ Institute for Integrative Biology of the Cell (I2BC) CEA, CNRS, Univ. Paris‐ Sud, Université Paris‐Saclay, Gif‐sur‐Yvette cedex Paris France

## Abstract

To explore the diversity of mobile genetic elements (MGE) associated with archaea of the phylum Thaumarchaeota, we exploited the property of most MGE to integrate into the genomes of their hosts. Integrated MGE (iMGE) were identified in 20 thaumarchaeal genomes amounting to 2 Mbp of mobile thaumarchaeal DNA. These iMGE group into five major classes: (i) proviruses, (ii) casposons, (iii) insertion sequence‐like transposons, (iv) integrative‐conjugative elements and (v) cryptic integrated elements. The majority of the iMGE belong to the latter category and might represent novel families of viruses or plasmids. The identified proviruses are related to tailed viruses of the order *Caudovirales* and to tailless icosahedral viruses with the double jelly‐roll capsid proteins. The thaumarchaeal iMGE are all connected within a gene sharing network, highlighting pervasive gene exchange between MGE occupying the same ecological niche. The thaumarchaeal mobilome carries multiple auxiliary metabolic genes, including multicopper oxidases and ammonia monooxygenase subunit C (AmoC), and stress response genes, such as those for universal stress response proteins (UspA). Thus, iMGE might make important contributions to the fitness and adaptation of their hosts. We identified several iMGE carrying type I‐B CRISPR‐Cas systems and spacers matching other thaumarchaeal iMGE, suggesting antagonistic interactions between coexisting MGE and symbiotic relationships with the ir archaeal hosts.

## Introduction

Similar to bacteria and eukaryotes, archaea are associated with diverse classes of mobile genetic elements (MGE), collectively referred to as the mobilome. Based on genomic features and the mode of interaction with the host cells, the archaeal mobilome can be divided into five large classes: (i) viruses (Pietilä *et al*., 2014; Snyder *et al*., 2015; Prangishvili *et al*., 2017; Krupovic *et al*., 2018; Munson‐McGee *et al*., 2018), (ii) conjugative elements (Prangishvili *et al*., 1998; Greve *et al*., 2004), (iii) small cryptic plasmids (Forterre *et al*., 2014; Wang *et al*., 2015), (iv) transposable elements closely related to bacterial insertion sequences (IS) (Filée *et al*., 2007) and (v) the more recently discovered self‐synthesizing transposon‐like elements called casposons which employ a homologue of the CRISPR‐associated Cas1 protein as their integrase (casposase) (Krupovic *et al*., 2014; Krupovic *et al*., 2017). All five classes of MGE are also represented in bacteria, whereas eukaryotes lack conjugative elements and casposons.

Viruses infecting archaea are notoriously diverse in terms of their virion morphologies and gene contents (Pietilä *et al*., 2014; Wang *et al*., 2015; Prangishvili *et al*., 2017; Krupovic *et al*., 2018; Munson‐McGee *et al*., 2018). Comparative structural and genomic studies show that the archaeal virosphere can be generally divided into two large fractions, the archaea‐specific viruses and the cosmopolitan viruses (Iranzo *et al*., 2016b). The archaea‐specific viruses are, by definition, unique to archaea and often display unexpected virion morphologies, such as bottle‐shaped, spindle‐shaped or droplet‐shaped (Prangishvili *et al*., 2017). Most of these viruses are, thus far, known to infect hyperthermophiles of the phylum Crenarchaeota. Archaea‐specific viruses are currently classified into 13 families that are characterized by unique gene contents that are distinct from those of viruses infecting bacteria and eukaryotes, and only minimally shared across different archaeal virus families. By contrast, the cosmopolitan fraction of the archaeal virosphere consist of viruses that display common architectural and genomic features with viruses of bacteria and eukaryotes, and for many genes, homologues in bacterial viruses are readily detectable (Iranzo *et al*., 2016b). These include tailed dsDNA viruses representing all three major families of the order *Caudovirales* (*Myoviridae*, *Siphoviridae* and *Podoviridae*), the dominant supergroup of bacterial viruses, as well as icosahedral viruses with the double jelly‐roll (DJR) and single jelly‐roll (SJR) major capsid proteins (MCP) classified in the families *Turriviridae* and *Sphaerolipoviridae*, respectively (Pietilä *et al*., 2014; Prangishvili *et al*., 2017).

Representatives of all five classes of archaeal (and bacterial) MGE can integrate into the genomes of their hosts and reside as integrated MGE (iMGE). In fact, a substantial fraction of cellular genomes, across all three domains of life, consists of diverse classes of iMGE (Craig *et al*., 2015). Very often, iMGE are not merely silent passengers within the cellular genomes but can have pronounced effects on the functioning, adaptation and evolution of their host cells. In bacteria, many adaptive traits, such as various transporters, antibiotic resistance genes or toxins, are encoded by integrative‐conjugative elements (ICE), pathogenicity islands and transposons which allow host bacteria to compete with other organisms for resources and colonize new ecosystems (Escudero *et al*., 2015; Johnson and Grossman, 2015; Guédon *et al*., 2017; Novick and Ram, 2017; Partridge *et al*., 2018). Indeed, pathogenicity determinants typically are carried by integrated or extrachromosomal MGE. Thus, the perception of iMGE as ‘junk DNA’ or ‘genomic parasites’ is changing to the concept of iMGE being major agents of molecular innovation and environmental adaptation of cellular organisms (Omelchenko *et al*., 2005; Frost and Koraimann, 2010; Frank and Feschotte, 2017; Jangam *et al*., 2017; Koonin and Krupovic, 2018). Typically, MGE integration leaves a molecular scar in the cellular genome which manifests as direct repeats (DR) flanking the iMGE (Grindley *et al*., 2006). In the case of integration mediated by tyrosine recombinases, the DR, known as left and right attachment sites (*attL* and *attR*), result from recombination between homologous sites present on the cellular chromosome and the MGE (Grindley *et al*., 2006). By contrast, the DR flanking transposons, as in the case of the recently described thaumarchaeal casposons (Krupovic *et al*., 2014; 2017), are referred to as target site duplication (TSD) and are generated by staggered cleavage of the target site, followed by fill‐in DNA repair (Mahillon and Chandler, 1998; Béguin *et al*., 2016).

Considerable efforts have been undertaken to explore the diversity and distribution of MGE in bacterial genomes. By contrast, our understanding of the archaeal mobilome remains limited. The vast majority of archaeal viruses and plasmids have been characterized from hyperthermophiles of the phylum Crenarchaeota and halophiles of the phylum Euryarchaeota (Forterre *et al*., 2014; Pietilä *et al*., 2014; Wang *et al*., 2015; Prangishvili *et al*., 2017; Munson‐McGee *et al*., 2018), whereas not a single virus or plasmid has been characterized for members of the third major phylum of cultivated archaea, the Thaumarchaeota. Thaumarchaea are among the most widely distributed archaea in the environment and are generally recognized to exert the primary control over ammonia oxidation in terrestrial, marine and geothermal habitats (Stahl and de la Torre, 2012). Due to their unusually high affinity for ammonia, this group of archaea is believed to outcompete the bacterial ammonia oxidizers in accessing ammonia and appear to determine the oxidation state of nitrogen available to associated microbial communities (Martens‐Habbena *et al*., 2009). Furthermore, as autotrophs, thaumarchaea also play an important role in the fixation of inorganic carbon. For instance, in oxygenated surface deep‐sea sediments, chemosynthesis largely depends on the oxidation of ammonia, with 1 mol of CO_2_ fixed per 10 mol of NH_4_^+^ oxidized (Wuchter *et al*., 2006).

It has been demonstrated that virus‐mediated turnover of thaumarchaea in surface deep‐sea sediments accounts for up to one‐third of the total microbial biomass killed, resulting in the release of approximately 0.3–0.5 gigatons of carbon per year globally and that turnover of thaumarchaea by viruses in the deep ocean is faster than that of bacteria (Danovaro *et al*., 2016). These findings illuminate the prominent role of thaumarchaeal viruses in the Biosphere (Danovaro *et al*., 2017). Despite the importance of thaumarchaea and their viruses in the global nitrogen and carbon cycling (Offre *et al*., 2013), only two proviruses (Krupovic *et al*., 2011; Abby *et al*., 2018) and three casposons (Krupovic *et al*., 2014; Krupovic *et al*., 2016) have been identified in the thaumarchaeal genomes. In addition, several putative thaumarchaeal virus genomes, all members of the order *Caudovirales*, have been sequenced in the course of single‐cell genomics and metagenomics studies (Chow *et al*., 2015; Labonté *et al*., 2015; Ahlgren *et al*., 2019; López‐Pérez *et al*., 2018), although metagenomics analyses have further hinted at an unexplored diversity of thaumarchaeal viruses (Danovaro *et al*., 2016; Roux *et al*., 2016; Vik *et al*., 2017). Furthermore, it is currently unclear whether any of the many morphologically unique viruses discovered in crenarchaea (Prangishvili *et al*., 2017) are associated with mesophilic archaea, such as thaumarchaea.

Here, we report the results of a search of the genomes of thaumarchaea isolated from diverse environments for iMGE. The identified iMGE are assigned to five classes, namely, proviruses, casposons, IS‐like transposons, putative integrative‐conjugative elements and cryptic integrated elements, and provide insights into the prevalence, diversity and distribution of the thaumarchaeal mobilome.

## Results

### *iMGE detection in thaumarchaeal genomes*

The genomes of 21 species representative of the taxonomic diversity and environmental distribution of the phylum Thaumarchaeota were analysed for the presence of iMGE (Supporting Information Table S1). The analysed genomes belong to four thaumarchaeal orders, namely, Cenarchaeales, Nitrosopumilales, Nitrososphaerales and *Candidatus* Nitrosocaldales, as well as four proposed unassigned genera, including *Ca*. Nitrosotalea, *Ca*. Nitrosotenuis, *Ca*. Nitrosopelagicus and *Ca*. Caldiarchaeum. The latter genus includes a single representative, *Ca*. Caldiarchaeum subterraneum, which in phylogenetic analyses forms a sister group to Thaumarchaeota and is usually assigned to a distinct archaeal phylum, the Aigarchaeota (Nunoura *et al*., 2011). However, in the GenBank database it is affiliated to the phylum Thaumarchaeota and was, thus, retained in our analysis. The analysed organisms were isolated from a wide range of environments, including a subsurface gold mine (Nunoura *et al*., 2011), thermal springs (Spang *et al*., 2012; Lebedeva *et al*., 2013; Abby *et al*., 2018; Daebeler *et al*., 2018), wastewater treatment plant (Li *et al*., 2016), marine waters (Santoro *et al*., 2015; Bayer *et al*., 2016; Ahlgren *et al*., 2017) and sediments (Park *et al*., 2014) and various soil samples (Kim *et al*., 2011; Lehtovirta‐Morley *et al*., 2011; Tourna *et al*., 2011; Zhalnina *et al*., 2014; Lehtovirta‐Morley *et al*., 2016; Herbold *et al*., 2017). Although most of these organisms are mesophiles, some are psychrophilic (Hallam *et al*., 2006), thermophilic (Nunoura *et al*., 2011; Spang *et al*., 2012; Lebedeva *et al*., 2013; Abby *et al*., 2018; Daebeler *et al*., 2018) or acidophilic (Lehtovirta‐Morley *et al*., 2011).

**Figure 1:**
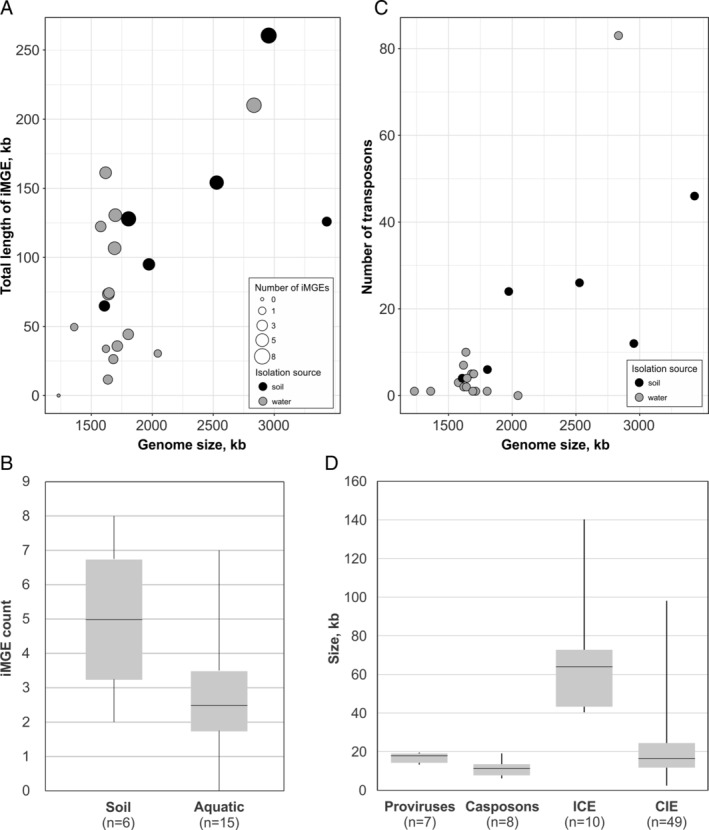
Characteristics of thaumarchaeal iMGE. A. Correspondence between the cumulative size of the iMGEs in the genome and the total genome size. Grey and black circles represent iMGEs present in the genomes of thaumarchaea isolated from aquatic and soil samples, respectively, with the diameter of the circles corresponding to the number of iMGEs per genome. B. Box plot shows the frequency of iMGE in genomes of thaumarchaea isolated from soil and aquatic (marine and freshwater) environments. C. Correspondence between the number of IS‐like transposons in the genome and the total genome size. Grey and black circles denote the IS identified in the genomes of thaumarchaea isolated from aquatic and soil samples, respectively. D. Box plot show size distribution in the four iMGE classes. Each box represents the middle 50th percentile of the data set and is derived using the lower and upper quartile values. The median value is displayed by a horizontal line inside the box. Whiskers represent the maximum and minimum values.

We employed three different strategies to search for the iMGEs (see Materials and Methods for details). Specifically, the genomes were analysed for the presence of (i) loci enriched in ORFans and uncharacterized genes; (ii) genes encoding signature proteins typical of different archaeal MGE groups; (iii) genes encoding integrases of the tyrosine recombinase superfamily. For detailed analysis and annotation, we considered only those loci that displayed typical features of site‐specific integration and/or contained signature MGE genes surrounded by additional virus‐ or plasmid‐related genes. In total, 74 iMGEs were predicted with high confidence in 20 thaumarchaeal genomes (Supporting Information Table S2), with the number of iMGE per genome ranging from 1 to 8 (median = 3). Only one of the analysed thaumarchaeal species, *Ca*. Nitrosopelagicus brevis CN25 (Santoro *et al*., 2015), lacked identifiable iMGEs. In addition to the multigene iMGE, 20 of the 21 analysed thaumarchaeal genomes were found to contain transposons closely related to bacterial insertion sequences (IS) (Mahillon and Chandler, 1998; Filée *et al*., 2007). The number of IS‐like transposons per genome varied from 0 in *Cenarchaeum symbiosum* A to 83 in *Ca*. Nitrososphaera gargensis Ga9_2 (Supporting Information Table S1). Thaumarchaea isolated from soil samples generally have larger genomes (*p* value = 0.093) and more iMGE per genome (*p* value = 0.072) than those inhabiting aquatic environments (Fig. 1A), whereas freshwater and marine thaumarchaea have similar numbers of iMGE. Consistently, *Ca*. Nitrosopelagicus brevis CN25, which does not carry identifiable iMGE, has the smallest genome (1.23 Mbp) among the sequenced thaumarchaea. Thus, the number of iMGE appears to scale close to linearly with the host genome size although, given the limited dataset, the two values show relatively weak positive correlation (*R* = 0.469, *p* value = 0.031; Fig. 1B). The number of the more abundant IS‐like transposons showed stronger correlation with the genome size (*R* = 0.738, *p* value = 0.00013; Fig. 1C). No statistically significant differences were observed in the number of iMGE or transposons between mesophiles and thermophiles.

### *Targets and molecular features of MGE integration*

**Figure 2:**
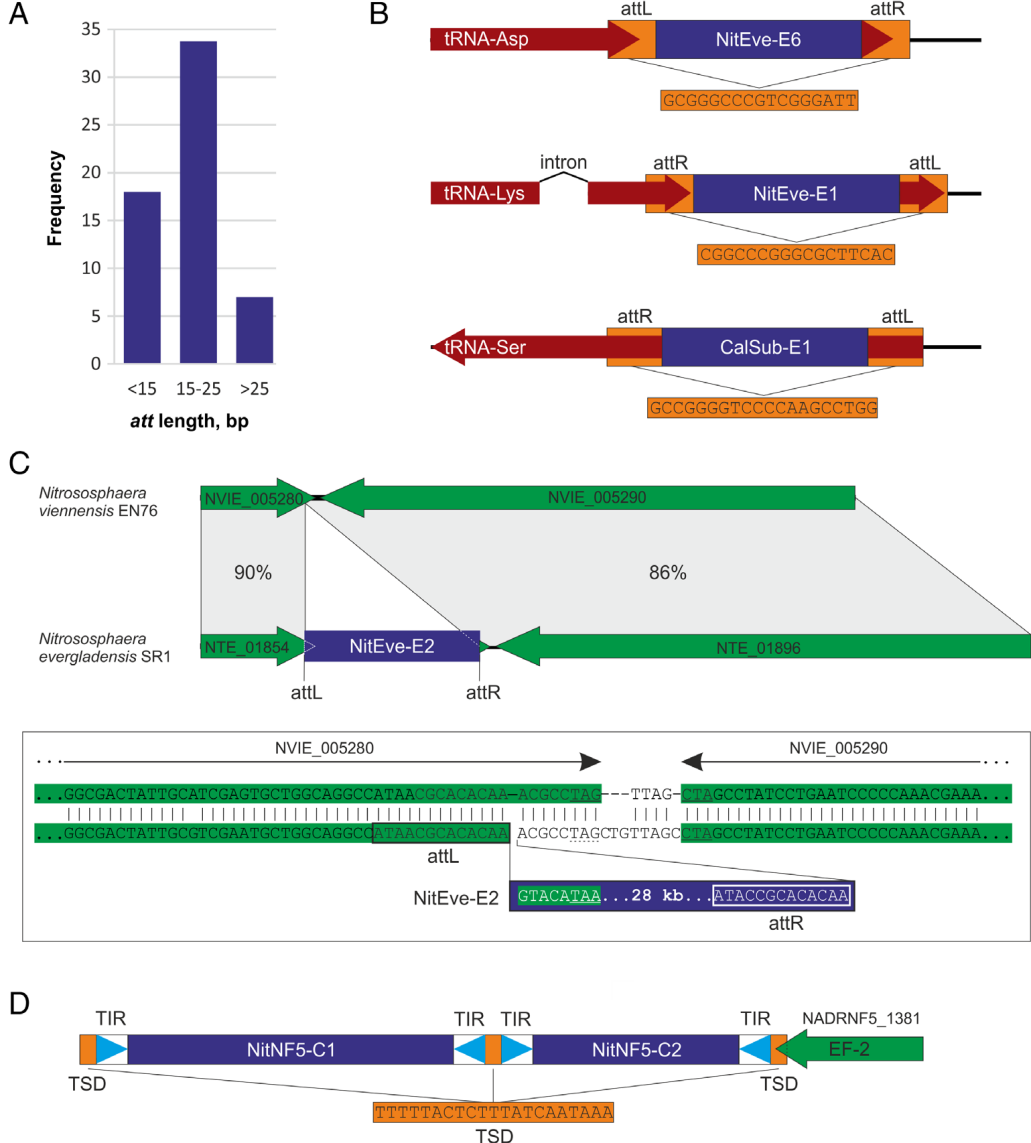
Properties of site‐specific MGE integration in thaumarchaea. A. Frequency of iMGE integration in different target sites. B. Integrations in tRNA genes. iMGE are indicated by blue rectangles; tRNA genes are shown as red arrows; attachment (att) sites are highlighted in orange. C. Integrations in protein‐coding genes. The protein coding genes are shown with green arrows, whereas the iMGE is shown as a blue rectangle. The figure compares an empty site in the genome of *Nitrososphaera viennensis* EN76 and an iMGE‐occupied site in the genome of *Nitrososphaera evergladensis* SR1. The box shows a zoom‐in on the corresponding integration sites in the two species. The original stop codon is underlined, whereas the one introduced by the iMGE is indicated with a broken line. Attachment sites are boxed. D. Tandem integration of two casposons into a protein‐coding gene. Terminal inverted repeats (TIR) are shown with light blue triangles, whereas target site duplications (TSD) are shown as orange rectangles. [Color figure can be viewed at wileyonlinelibrary.com]

The putative *att*/TSD sites could be determined for 68 of the 74 elements (Supporting Information Table S2). Of the six iMGE for which *att*/TSD could not be confidently predicted, five are proviruses and one is a cryptic integrated element. These might be either inactivated iMGE or their recombination sites could be too short for unambiguous identification without additional sequence information from closely related strains. The DR flanking the thaumarchaeal elements were considerably shorter than those characteristic of iMGEs from other archaea. The majority of thaumarchaeal *att* sites were shorter than 26 bp (as short as 8 bp, median length of 17 bp); only seven iMGEs had *att* sites longer than 25 bp (Fig. 2A). By contrast, the *att* sites characterized for MGEs integrated in crenarchaeal genomes ranged from 29 to 69 bp (median length of 45 bp) (She *et al*., 2002). Similarly to the case of bacteria, archaeal MGEs often integrate into tRNA genes (Williams, 2002; She *et al*., 2004; Krupovic *et al*., 2010b; Béguin *et al*., 2016; Cossu *et al*., 2017; Wang *et al*., 2018a). However, other integration targets, including protein‐coding genes and intergenic regions, have also been reported (Krupovic *et al*., 2010a; 2014; Shah *et al*., 2012;Anderson *et al*., 2017). Among the 68 thaumarchaeal iMGEs for which precise integration sites could be defined, 39 used tRNA genes as integration targets, 15 were found in the intergenic regions and 14 integrated into the 3′‐distal regions of protein‐coding genes (Supporting Information Table S2). There was no apparent relationship between the type of integration target used and the host organism or the type of iMGE. Several thaumarchaea hosted iMGEs which occupied all three types of target sites within the same genome (Supporting Information Table S2).

#### *Integration into tRNA genes*

Thirty‐nine iMGE integrations (57%) were identified in genes encoding tRNAs with 22 anticodons corresponding to 14 amino acids (Supporting Information Table S2). Notably, insertions occurred within both intron‐less (*n* = 29) and intron‐containing (*n* = 10) tRNA genes (Fig. 2B). *Ca*. Nitrosotalea okcheonensis CS contained four different elements integrated in distinct tRNA genes, whereas in *Ca*. Nitrosotenuis sp. AQ6f, four tRNA genes accommodated five different elements.

In bacteria and archaea, MGEs targeting tRNA genes typically recombine with the 3′‐distal region of the gene (Williams, 2002; She *et al*., 2004), whereas recombination with the 5′‐distal region is considerably less frequent (Zhao and Williams, 2002; Krupovic and Bamford, 2008b; Krupovic *et al*., 2010b; Gaudin *et al*., 2014; Cossu *et al*., 2017). All but one thaumarchaeal tRNA‐targeting iMGEs were found to be integrated into 3′‐distal regions of the tRNA genes. However, in the genome of *Ca*. C. subterraneum, CalSub‐E1 apparently recombined with the 5′‐distal region of the tRNA‐Ser gene (Fig. 2B).

*Ns. evergladensis* SR1 genome carries a curious chimeric iMGE that appears to result from integration of a smaller element, NitEve‐E7, into the genome of a larger one, NitEve‐E6. The latter is inserted into a tRNA gene, whereas the integration site of the former element, in the absence of sequences from closely related species, could be defined only approximately. Such piggybacking might be particularly beneficial for MGEs that do not encode specialized devices for intercellular transfer (e.g. conjugative pili). Integration into other MGEs might ensure wider horizontal spread of such elements. This strategy of dissemination is indeed widely employed by various insertion sequences which commonly integrate into larger MGE and has also been observed for casposons in *Methanosarcina* (Krupovic *et al*., 2016). Notably, seven thaumarchaeal iMGE from four different species carry transposon insertions.

#### *Integration into protein‐coding genes*

Fourteen iMGEs used protein‐coding genes for integration. The genes that are exploited by the MGE as integration targets encode a Zn‐finger protein conserved in different species of *Nitrososphaera* (AIF83914), AsnC family transcriptional regulator (AFU58629), dihydroxy‐acid dehydratase (ABX12782), diphthamide biosynthesis protein (CUR52689), phosphoribosylamine‐glycine ligase (CUR51614), glucosamine‐1‐phosphate N‐acetyltransferase (WP_075054010), elongation factor 2 (WP_014964994, WP_014963048, WP_048116371, CUR52052) and several conserved hypothetical proteins (WP_014962442, AJM91735, AJM92436). Notably, the orthologous genes for hypothetical proteins in *Ca*. Nitrosopumilus piranensis D3C (AJM91735) and *Ca*. Np. koreensis AR1 (WP_014962442) are targeted by two unrelated iMGEs, whereas in *Np. maritimus* SCM1 and *Ca*. Np. adriaticus NF5, the corresponding genes are free of MGE integrations.

Due to the fact that *att*/TSD sites of thaumarchaeal elements are generally short (Fig. 2A), their unambiguous identification was challenging, particularly when integration occurred within unorthodox targets such as protein‐coding genes. In all cases, the putative integration sites were meticulously verified by comparison of the corresponding genomic loci from closely related organisms with and without MGE insertions. An example of such analysis is shown in Fig. 2C. In the *Ns. evergladensis* SR1 genome, NitEve‐E2 is inserted into the 3′‐distal region of a gene encoding a Zn‐finger protein (AIF83914). Although, the predicted *att* site is only 13 bp‐long, comparison with the corresponding region in *Ns. viennensis* EN76 provided unequivocal support for the prediction site. Interestingly, NitEve‐E2 insertion replaced a eight nucleotide sequence of the target gene including the stop codon (TAG) with a non‐homologous MGE‐derived sequence which contains an alternative stop codon (TAA), reconstituting the open reading frame (Fig. 2C).

A gene encoding elongation factor 2 (EF‐2), a GTPase involved in the translocation step of the ribosome during protein synthesis, seems to serve as the most common target for integration of thaumarchaeal casposons (Krupovic *et al*., 2014). The integration of the casposons NitAR1‐C1 and NitAR2‐C1 in the genomes of *Ca*. Np. koreensis AR1 and *Ca*. Np. sediminis AR2, respectively, has been described previously (Krupovic *et al*., 2014). In the present study, we identified two new casposons, NitNF5‐C1 and NitNF5‐C2 (see below for description), which use the same cellular gene for integration, in the genome of *Ca*. Np. adriaticus NF5. The two elements are inserted in tandem into the same *ef‐2* gene (Fig. 2D). Such tandem integrations have been previously described in the case of family 2 casposons in *Methanosarcina* sp. (Krupovic *et al*., 2016), but have not been observed for thaumarchaeal family 1 casposons. Notably, archaeal and bacterial MGEs that use tyrosine recombinases for integration are also known to form arrays of integrated elements by re‐using the same integration site (Krupovic and Bamford, 2008b; Krupovic *et al*., 2010b; Das *et al*., 2013). *Ca*. Nt. devanaterra contains two family 1 casposons as well. One of these is also integrated in the *ef‐2* gene, whereas the other one is inserted into the 3′‐distal region of a gene encoding phosphoribosylamine‐glycine ligase. Finally, the NitEve‐C1 casposon identified in the *Ns. evergladensis* SR1 genome does not target any protein‐coding genes but is inserted into an intergenic region. These new observations indicate that *ef‐2* is not the universal target for thaumarchaeal casposons, even within the genus *Nitrosopumilus*.

### *Five major classes of thaumarchaeal MGE*

Based on the gene content analysis, the thaumarchaeal iMGE could be broadly grouped into five major classes: (i) proviruses, (ii) casposons, (iii) putative integrative‐conjugative elements (ICE), (iv) cryptic integrated elements (CIE) and (v) IS‐like transposons. The first four classes include complex, multigene mobile elements, whereas IS‐like transposons typically consist of 1 or 2 genes, one of which encodes a transposase. Hereafter, we reserve the term iMGE for the complex elements. The majority (*n* = 48) of the identified iMGE belong to the CIE category and might represent novel families of viruses or plasmids. The identified iMGE greatly vary in size, spanning nearly three orders of magnitude from 2.6 to 140 kb (median size of 16.8 kb; Fig. 1D). Collectively, the 74 iMGE amount to 1 938 724 bp of mobile thaumarchaeal DNA. Proviruses and casposons are rather uniform in size, all smaller than 20 kb, whereas ICE and CIE are more variable and reach 140 and 98 kb, respectively (Fig. 1D). Below we characterize all five classes of thaumarchaeal MGE in more detail.

#### *Proviruses*

**Figure 3:**
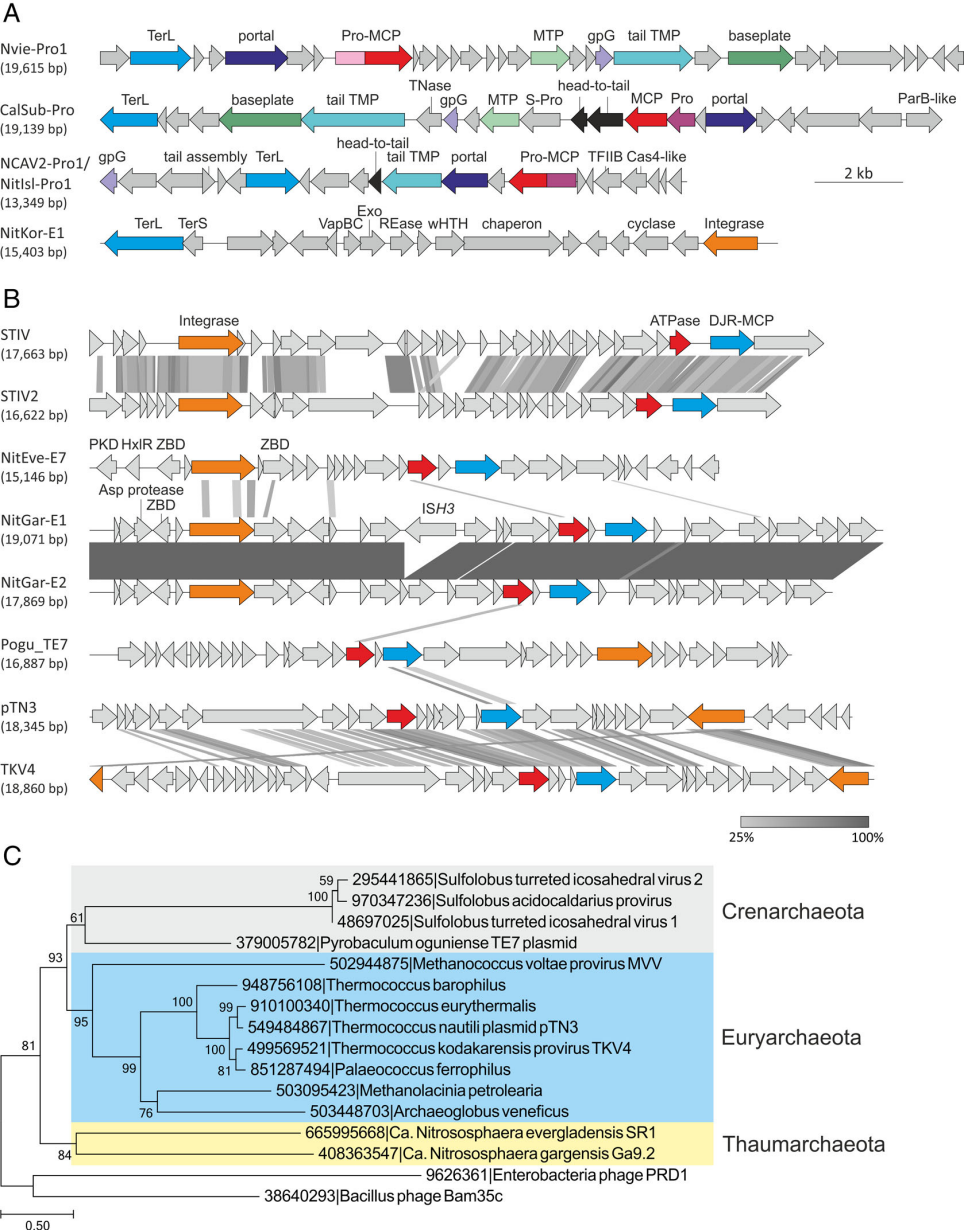
Comparison of thaumarchaeal proviruses. A. Genome maps of proviruses related to tailed bacterial and archaeal viruses of the order *Caudovirales*. Functionally equivalent genes are shown using the same colours. Abbreviations: TerS and TerL, small and large subunits of the terminase, respectively; Pro, prohead maturation protease; S‐Pro, serine protease; MCP, major capsid protein; MTP, major tail protein; TMP, tape measure protein; Exo, exonuclease; REase, restriction endonuclease; wHTH, winged helix‐turn‐helix. B. Genome maps of archaeal viruses and proviruses encoding the DJR MCPs. Functionally equivalent genes are shown using the same colours. Abbreviations: ATPase, A32‐like genome packaging ATPase; ZBD, zinc‐binding domain‐containing protein; HxlR, HxlR family DNA‐binding transcriptional regulator; PKD, PKD (Polycystic Kidney Disease) domain‐containing protein; IS*H3*, IS*H3* family insertion sequence. For more detailed annotation see Supporting Information data file 1. C. Maximum likelihood phylogeny of concatenated A32‐like ATPase and DJR‐MCP proteins. The tree was constructed using the automatic optimal model selection (RtREV +G6 + I + F) and is rooted with bacterial tectiviruses. The scale bar represents the number of substitution per site. [Color figure can be viewed at wileyonlinelibrary.com]

Two groups of putative proviruses were identified in thaumarchaeal genomes: proviruses related to tailed bacterial and archaeal viruses of the order *Caudovirales*, and those related to viruses encoding the double jelly‐roll (DJR) major capsid proteins (MCP). Searches initiated with the sequences of the large terminase subunit (TerL), a signature protein of the *Caudovirales*, yielded five hits in thaumarchaeal genomes. Two of these hits were to the previously reported putative proviruses Nvie‐Pro1 and NCAV2‐Pro1 in the genomes of *Ns. viennensis* EN76 (Krupovic *et al*., 2011) and *Ca*. Nitrosocaldus cavascurensis SCU2 (Abby *et al*., 2018), respectively. The three new hits were in the genomes of *Ca*. C. subterraneum, *Ca*. Np. koreensis AR1 and *Ca*. Nitrosocaldus islandicus 3F. The latter element was identical to NCAV2‐Pro1 from *Ca*. Nc. cavascurensis SCU2. In Nvie‐Pro1 and NCAV2‐Pro1, potential recombination sites and, consequently, the exact boarders of the elements could not be detected (Krupovic *et al*., 2011; Abby *et al*., 2018). Similarly, the boarders of CalSub‐Pro in the genome of *Ca*. C. subterraneum could be determined only approximately. However, analysis of the gene content in the vicinity of *terL* in Nvie‐Pro1, NCAV2‐Pro1 and CalSub‐Pro identify genes for all components necessary for the morphogenesis of full‐fledged tailed virions. In CalSub‐Pro, we identified gene homologues of the HK97‐like MCP, the portal protein as well as the major and minor tail proteins, including the baseplate, head to tail connector, tail tape measure and tail fibre proteins (Fig. 3A). CalSub‐Pro also contains a gene for the putative capsid maturation protease. Whereas Nvie‐Pro1 encodes a chymotrypsin‐like protease fused to the MCP (Krupovic *et al*., 2011), CalSub‐Pro carries a gene for the typical S78‐family caudoviral prohead protease (Pfam id: PF04586) located immediately upstream of the MCP gene, a typical gene order in *Caudovirales*. NCAV2‐Pro1 (and NitIsl‐Pro1) also encode a typical caudoviral prohead protease; however, unlike in CalSub‐Pro but similar to Nvie‐Pro1, the protease domain is fused to the MCP (Fig. 3A), highlighting the fluidity of the morphogenetic module in thaumarchaeal head‐tail proviruses. Interestingly, neither of the proviruses contains identifiable genes for genome replication proteins. Given the lack of identifiable *att* sites and genome replication apparatus, on the one hand, and the presence of an apparently functional virion morphogenesis module on the other hand, there is a distinct possibility that the corresponding loci represent domesticated *Caudovirales*‐derived elements, akin to the gene transfer agents (GTA) operating in some bacteria and euryarchaea (Lang *et al*., 2012; Lang *et al*., 2017; Koonin and Krupovic, 2018). Alternatively, these loci could be remnants of inactivated proviruses although conservation of the morphogenetic modules argues against this possibility. Notably, despite the shared gene contents, the three head‐tail virus‐derived elements described above are highly divergent and appear to be derived from distinct members of the *Caudovirales*.

Analysis of the *Ca*. Np. koreensis AR1 genome showed that the TerL homologue is indeed encoded within a putative iMGE, NitKor‐E1. However, the only other identifiable *Caudovirales*‐like gene in this elements was that for the small terminase subunit (TerS), located immediately upstream of the TerL‐encoding gene, a typical location for this gene. All other genes in this element, although typical of MGE, could not be attributed to *Caudovirales* or any other group of viruses and included a VapBC toxin‐antitoxin system, PD‐(D/E)XK family restriction endonuclease and tyrosine integrase (Fig. 3A). The terminase complex is highly specific to viruses of the orders *Caudovirales* and *Herpesvirales*, and so far has not been identified in nonviral MGE. Thus, its function in NitKor‐E1 remains enigmatic but likely is a relic from a past integration of a head‐tailed virus. However, in the absence of other viral signature genes and experimental evidence of virion formation, we classify NitKor‐E1 as a CIE rather than a provirus.

Viruses with the DJR MCPs infect hosts in all three domains of life (Krupovic and Bamford, 2008a; Krupovic and Koonin, 2015). In addition to the DJR MCP, these viruses share a specific genome packaging ATPase of the FtsK‐HerA superfamily (Iyer *et al*., 2004) which is unrelated to TerL proteins of *Caudovirales* and *Herpesvirales*. The genes for the capsid protein and the packaging ATPases are typically encoded close to each other and appear to be inherited as a single module. In archaea, this supergroup of viruses is represented by *Sulfolobus* turreted icosahedral viruses, STIV and STIV2, two members of the family *Turriviridae* (Rice *et al*., 2004; Happonen *et al*., 2010). However, several other integrated and extrachromosomal MGE encoding both signature proteins have been described in euryarchaea and crenarchaea (Krupovic and Bamford, 2008b; Bernick *et al*., 2012; Gaudin *et al*., 2014; Rensen *et al*., 2015). The viral nature of these MGE has not been confirmed. However, a provirus closely related to STIV and STIV2 is integrated in the genome of certain *S. acidocaldarius* strains (Anderson *et al*., 2017; Mao and Grogan, 2017), suggesting that the euryarchaeal iMGE also represent functional viruses. Recently, homologues of DJR MCP have been reported also in thaumarchaea, but the exact boundaries of the putative proviruses have not been defined (Yutin *et al*., 2018). Searches seeded with the sequence of the STIV MCP yielded hits to three proteins in thaumarchaea: two identical proteins are encoded in the genome of *Ca*. Ns. gargensis Ga9_2 and the third one in the genome of *Ca*. Ns. evergladensis SR1.

The two identical MCP homologues in *Ca*. Ns. gargensis Ga9_2 genome are encoded within two nearly identical proviruses, NitGar‐E1 and NitGar‐E2, tandemly integrated into the same target site within an intergenic region. The most notable difference between the two elements is the presence of an IS*H3* family insertion sequence in NitGar‐E1 (Fig. 3B). NitEve‐E7 of *Ca*. Ns. evergladensis SR1 is only distantly related to the proviruses of Ns. gargensis Ga9_2. As aforementioned, NitEve‐E7 is integrated into NitEve‐E6, an integrative‐conjugative element (see below), suggesting that NitEve‐E7 piggybacks NitEve‐E6 to be transferred between cells via conjugation. Genomic context analysis shows that the MCP genes are encoded in the vicinity of a predicted genome packaging ATPases, as is the case for other archaeal viruses and proviruses of this supergroup (Fig. 3B). Besides the MCP and ATPase, the proviruses also share divergent integrases of the tyrosine recombinase superfamily. To better understand the evolutionary relationships among archaeal DJR MCP‐encoding proviruses, we constructed a maximum likelihood phylogeny of concatenated ATPase and MCP proteins, two signature proteins shared by all elements, from representative (pro)viruses associated with crenarchaea, euryarchaea and thaumarchaea. Note that although all proviruses also encode integrases, these do not appear to be orthologous and seem to have been independently acquired or replaced in different virus lineages. The phylogenetic tree rooted with bacterial tectiviruses revealed three clades corresponding to 3 different archaeal phyla, Crenarchaeota, Euryarchaeota and Thaumarchaeota, respectively (Fig. 3C). This result suggests deep association and co‐evolution of DJR MCP‐encoding viruses with their archaeal hosts or distinct origins of these viruses in different archaeal phyla. Many more representatives of this virus supergroup from different archaeal phyla will be needed to distinguish between the two possibilities.

#### *Casposons*

**Figure 4:**
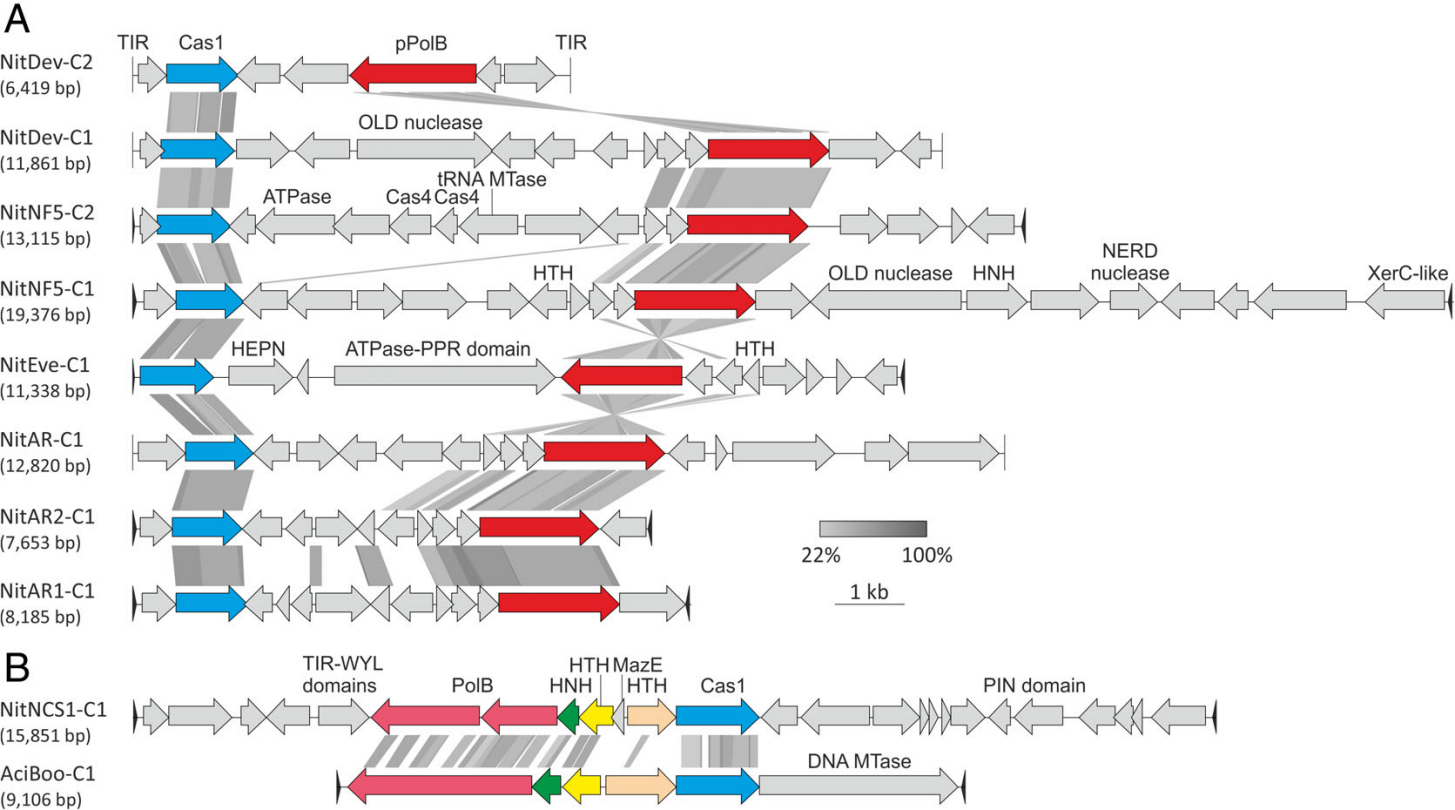
Comparison of thaumarchaeal casposons. A. Family 1 casposons. B. Comparison of the family 2 casposons from *Ca*. Nitrosotalea okcheonensis CS (NitNCS1‐C1) and *Aciduliprofundum boonei* (AciBoo‐C1). Homologous genes are shown using the same colours. Abbreviations: TIR, terminal inverted repeats; (p)PolB, (protein‐primed) family B DNA polymerase; OLD, OLD (overcome lysogenization defect) family nuclease; HTH, helix‐turn‐helix; HNH, HNH family nuclease; MTase, methyltransferase. For detailed annotation see Supporting Information data file 1. [Color figure can be viewed at wileyonlinelibrary.com]

Previously, we described 3 distinct thaumarchaeal casposons which were classified into family 1 (Krupovic *et al*., 2014). Differently from casposons from families 2, 3 and 4, family 1 casposons encode family B DNA polymerases (PolB) that shows the closest sequence similarity to protein‐primed PolBs (pPolB) of archaeal viruses (Krupovic *et al*., 2014). Here, we identified five distinct family 1 casposons in the genomes of *Ca*. Ns. evergladensis SR1, *Ca*. Np. adriaticus NF5 and *Ca*. Nt. devanaterra. The latter two species each contain two casposons. Whereas the two casposons in *Ca*. Np. adriaticus NF5 are tandemly integrated into the same target site (Fig. 2D), those in *Ca*. Nt. devanaterra are inserted into different protein‐coding genes. Notably, the five casposons are not closely related to each other or to those described previously (Fig. 4A).

Besides the genes for Cas1 and pPolB, family 1 casposons share 3 or 4 uncharacterized genes encoded immediately upstream of the *pPolB* gene. In addition, each casposon carries element‐specific genes (Fig. 4A). The new casposons encode several nucleases that have not been previously observed in family 1, including OLD family nucleases (in NitDev‐C1 and NitNF5‐C1), NERD domain‐containing nuclease related to Holliday junction resolvases (NitNF5‐C1) and HNH nuclease (NitNF5‐C1). Most notably, NitNF5‐C2 encodes two homologues of the Cas4 nuclease, which is involved in the adaptation process in many CRISPR‐Cas systems (Hudaiberdiev *et al*., 2017; Kieper *et al*., 2018; Lee *et al*., 2018; Shiimori *et al*., 2018), and might participate in casposon integration, which is mechanistically closely similar to CRISPR spacer integration (Béguin *et al*., 2016; Krupovic *et al*., 2017). Both Cas4 copies display closest sequence similarity to Cas4 homologues from different *Clostridia*. Furthermore, NitEve‐C1 encodes a HEPN nuclease, a member of an expansive nuclease family that is typically associated with various microbial defence systems, including toxin‐antitoxin, abortive infection, restriction‐modification as well as type III and type VI CRISPR‐Cas systems (Anantharaman *et al*., 2013; Shmakov *et al*., 2015).

Finally, we identified a new casposon, NitNCS1‐C1, in *Ca*. Nitrosotalea okcheonensis CS, which does not belong to family 1. It shares highest sequence similarity to the family 2 casposon AciBoo‐C1 from *Aciduliprofundum boonei* (phylum Euryarchaeota), the only experimentally studied casposon thus far (Hickman and Dyda, 2015; Béguin *et al*., 2016). NitNCS1‐C1 encodes a conserved set of proteins typical of family 2 casposons, including a distinct PolB, Cas1, HNH nuclease and 2 helix‐turn‐helix proteins (Fig. 4B). Notably, it also encodes a protein containing a WYL domain that is often found in regulators of the CRISPR‐Cas systems (Makarova *et al*., 2014b; Yan *et al*., 2018). The PolB gene of NitNCS1‐C1 appears to be fragmented, and it remains unclear whether the two fragments constitute a functional protein or the element is inactivated. Similar to AciBoo‐C1 but unlike all other thaumarchaeal casposons, NitNCS1‐C1 is inserted into a tRNA‐Pro gene. Accordingly, NitNCS1‐C1 is the first family 2 casposon in Thaumarchaeota.

#### *Integrative‐conjugative elements*

**Figure 5:**
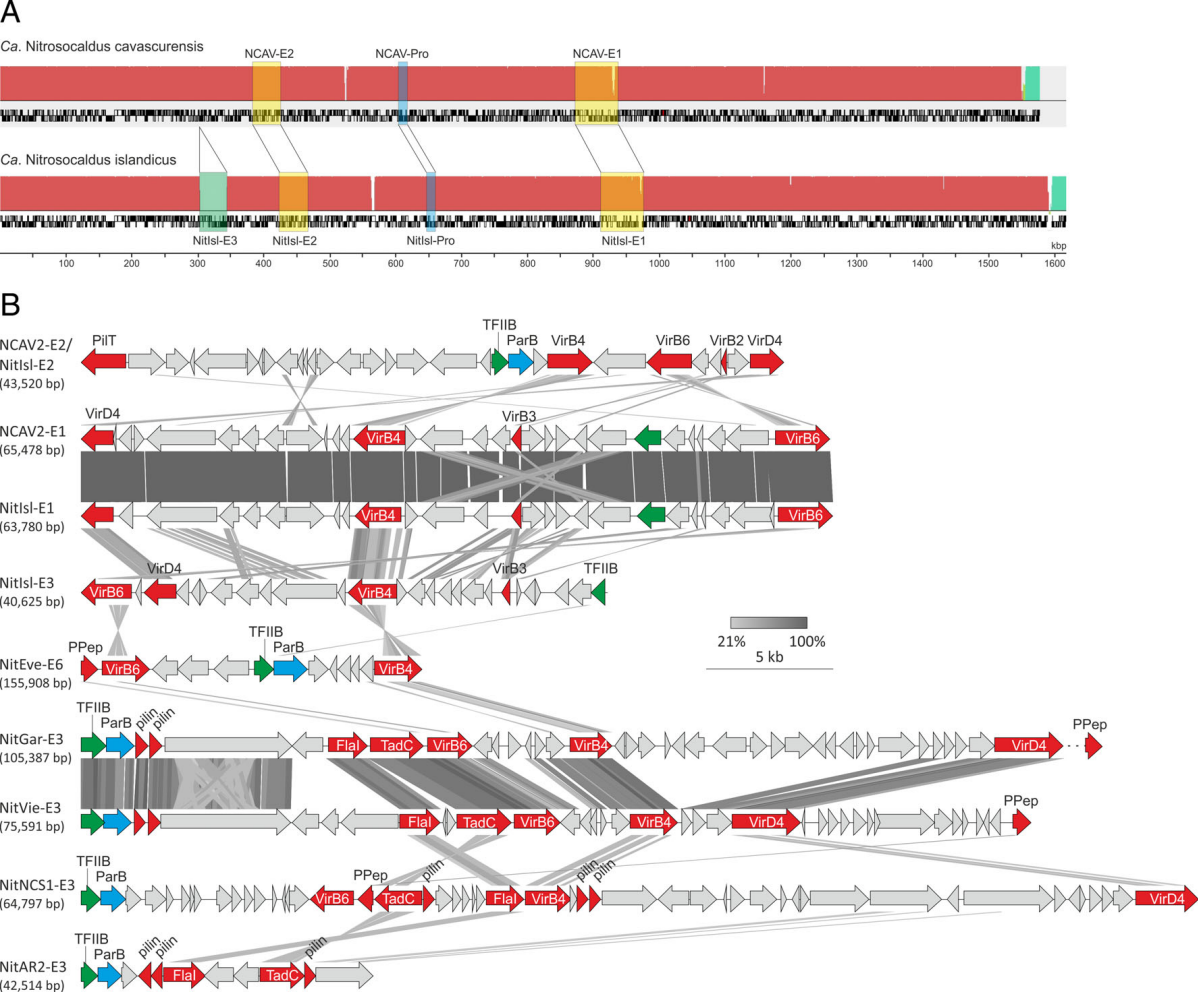
Comparison of thaumarchaeal integrative‐conjugative elements. A. Comparison of the genomes of two closely related *Nitrosocaldus* strains, *Ca*. Nc. cavascurensis SCU2 and *Ca*. Nc. islandicus 3F. Shared ICEs and proviruses are indicated with transparent yellow and blue boxes, whereas the ICE element unique to *Ca*. Nc. islandicus 3F is shown highlighted with a green box. B. Thaumarchaeal integrative‐conjugative elements. Only regions including the genes encoding the predicted components of the conjugation apparatus are depicted (highlighted in red). Genes for the ParB‐like segregation protein and TFIIB transcription initiation factor are shown in blue and green, respectively. PPep, prepilin peptidase. For detailed annotation see Supporting Information data file 1. [Color figure can be viewed at wileyonlinelibrary.com]

The third type of identified thaumarchaeal iMGE are potential ICEs. ICEs are the largest among the four iMGE categories (median size of 64 kb; Fig. 5A). Two ICEs, NCAV2‐E1 and NCAV2‐E2, have been recently described in the genome of *Ca*. Nc. cavascurensis SCU2 (Abby *et al*., 2018). Here, we identified eight additional ICEs (Supporting Information Table S2). Similar to NCAV2‐Pro1, orthologs of NCAV2‐E1 and NCAV2‐E2 are present in the genome of a closely related (ANI = 99.9%) species *Ca*. Nc. islandicus 3F (Daebeler *et al*., 2018). Notably, however, *Ca*. Nc. islandicus 3F harbours an additional ICE, NitIsl‐E3, compared to *Ca*. Nc. cavascurensis SCU2, which instead has an empty site (Fig. 5A), confirming the recent mobility of NitIsl‐E3. Figure 5B shows the regions of thaumarchaeal ICEs containing genes encoding components of the predicted conjugation/secretion systems. Similar to conjugative plasmids of *Sulfolobus* (Prangishvili *et al*., 1998; Greve *et al*., 2004), most of the thaumarchaeal ICEs carry a pair of signature genes for the homologues of VirB4/TrbE and VirD4/TraG ATPases which energize type IV secretion systems (Wallden *et al*., 2010). Other conserved components include homologues of the integral membrane proteins VirB6, VirB3 and TadC; FlaI and PilT ATPases; prepilin peptidase and pilins (Fig. 5B). Furthermore, all identified thaumarchaeal ICEs encode homologues of transcription factor IIB (TFIIB) which, in most elements, are located immediately upstream of the genes for the ParB‐like partitioning protein, likely, in the same operon. Notably, TFIIB homologues have been previously detected in the vicinity of genes encoding type IV secretion systems in other archaea (Makarova *et al*., 2016). However, coupling with ParB appears to be specific to thaumarchaeal ICEs. Overall, the conserved genes were not syntenic (except in the orthologous ICEs; Fig. 5B), suggesting extensive recombination within the putative conjugation module. We did not detect candidates for relaxases which generate a single‐stranded copy of ICE DNA prior to transfer in bacteria (Johnson and Grossman, 2015). However, typical relaxases are also absent in the bona fide conjugative plasmids of *Sulfolobus*, consistent with the suggestion that the archaeal conjugation machinery is distinct from that of bacteria and might transfer dsDNA as the substrate (Greve *et al*., 2004).

The predicted DNA replication modules of the thaumarchaeal ICEs also show considerable differences. Only NitEve‐E6, the largest identified ICE, encodes its own DNA polymerase (PolB) that is more closely related to the PolBs from family 2 casposons (Krupovic *et al*., 2014) (hit to NitNCS1‐C1 casposon, *E* = 3e‐38, 41% identity), rather than to cellular replicative polymerases which were not recovered even after several PSI‐BLAST iterations. NitGar‐E3 and NitVie‐E3 encode homologues of the Cdc6/Orc1 replication initiator, whereas NitVie‐E3 and NitNCS1‐E3 encode UvrD‐like superfamily 1 helicases. NCAV2‐E2 (and orthologous NitIsl‐E2) carry genes for type IA topoisomerases which could also participate in their replication. NCAV2‐E1 (and orthologous NitIsl‐E1) and NitIsl‐E3 encode MGE‐specific replication proteins containing an N‐terminal archaeo‐eukaryotic primase (AEP) domain (also referred to as the primpol domain) and a C‐terminal superfamily 3 helicase (S3H) domain, an organization commonly found in replication proteins of various MGE and viruses (Iyer *et al*., 2005; Lipps, 2011; Kazlauskas *et al*., 2018). The diversity of genome replication modules associated with thaumarchaeal ICEs suggests distinct origins and evolutionary histories of these elements.

#### *Cryptic integrated elements*

**Figure 6:**
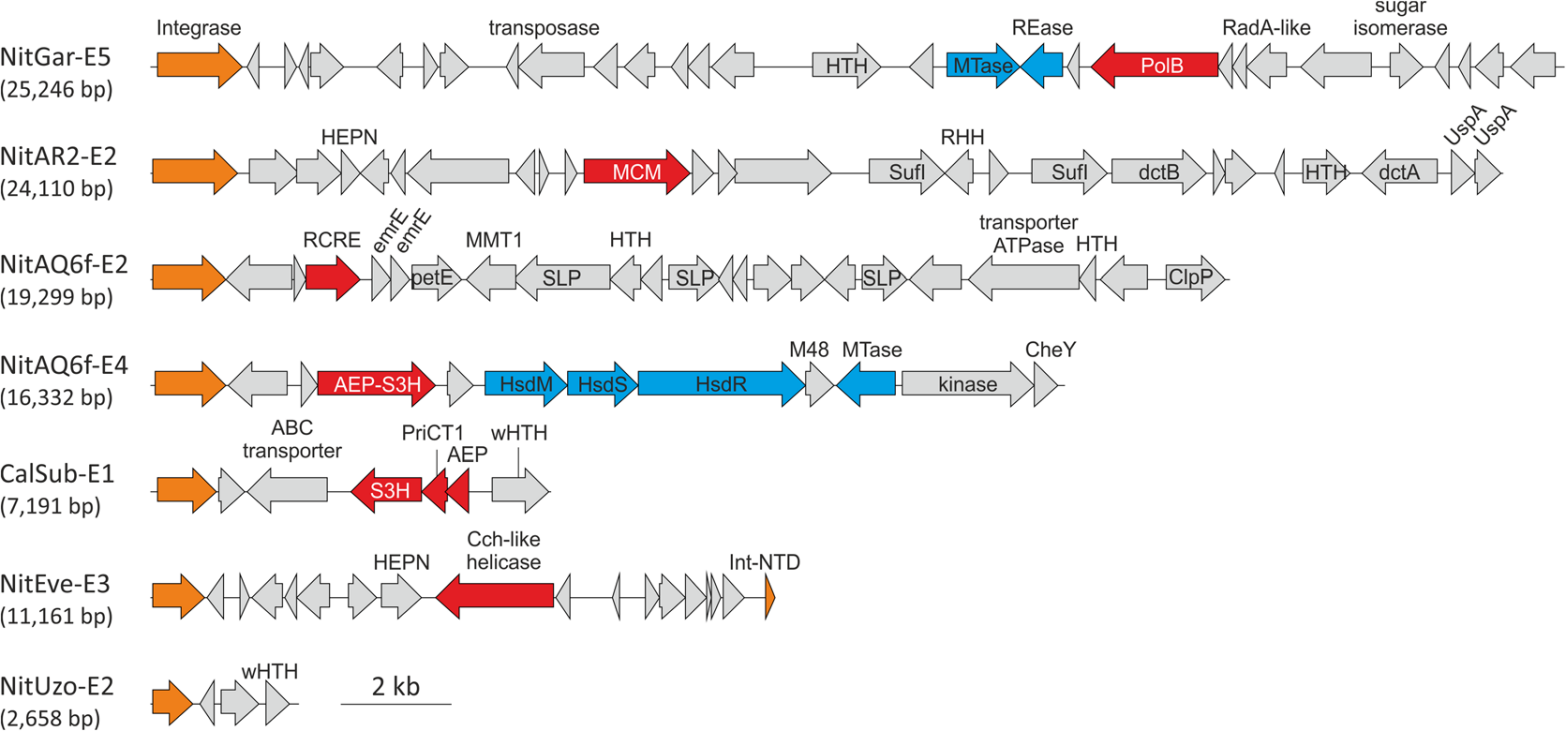
Genome maps of selected thaumarchaeal cryptic integrated elements. Integrase genes are highlighted in orange, gene encoding diverse replication‐associated proteins are shown in red and components of the restriction‐modification systems are in blue. Abbreviations: UspA, UspA family nucleotide‐binding protein; dctA, C4‐dicarboxylic acids transport protein (Na+/H+ dicarboxylate symporter); dctB, C4‐dicarboxylate transport sensor protein; SufI, multicopper oxidase; SLP, S‐layer protein with immunoglobulin domain; PetE, Plastocyanin/azurin/halocyanin family protein; MMT1, Co/Zn/Cd cation transporter; HsdM/S/R, type I restriction‐modification system methyltransferase/specificity/restriction subunits; MTase, methyltransferase; Mod: Adenine‐specific DNA methyltransferase; REase, restriction endonuclease; M48, M48 family peptidase; CheY, chemotaxis protein receiver domain; EmrE, membrane transporter of cations and cationic drugs; RHH, ribbon‐helix–helix domain‐containing protein; (w)HTH, (winged) helix‐turn‐helix; RCRE, rolling circle replication initiation endonuclease; AEP, archaeo‐eukaryotic primase; S3H, superfamily 3 helicase; MCM, minichromosome maintenance helicase. [Color figure can be viewed at wileyonlinelibrary.com]

The CIE vary in size from 2.6 kb to 98 kb but the majority are smaller than 20 kb (median = 17 kb; Fig. 1D). There are no discernible signature genes that would be specific to thaumarchaeal CIE. By definition, the most conserved protein, although belonging to different arCOGs, is the integrase. Interestingly, NitEve‐E3 encodes an SSV1‐like integrase which is split into two fragments upon integration of the MGE although no other homologues of viral genes were identified in this element. Similar to ICE, CIE encode diverse genome replication proteins, including those specific to MGEs (Fig. 6). ThaMY3‐E2, the largest of the identified CIE (98.3 kb), encodes homologues of PolB and archaeal replicative helicase MCM, whereas NitGar‐E6 and NitEve‐E3 encode MCM but not PolB. The MCM helicases have been previously found to be frequently recruited from the host as the main replication proteins of various crenarchaeal and euryarchaeal MGEs, including viruses and plasmids (Krupovic *et al*., 2010b; Kazlauskas *et al*., 2016). By contrast, NitDev‐E3 and NitAR2‐E2 encode a superfamily 2 helicase and a homologue of the Cch helicase (AAA+ ATPase superfamily) from a *Staphylococcus aureus* mobile genomic island (Mir‐Sanchis *et al*., 2016), respectively. NitAQ6f‐E1 encodes a homologue of the Cdc6/Orc1 replication initiator, a distant homologue of the MCM helicases. Presumably, both the MCM helicases and Orc1 recruit the cellular replisome for the MGE replication. Some CIE, such as CalSub‐E1, NitKor_MY1‐E1 and NitAQ6f‐E4, encode primpols. In the corresponding NitKor_MY1‐E1 and NitAQ6f‐E4 proteins, the primpol domain is fused to the S3H domain. By contrast, in CalSub‐E1, the primpol domain, the α‐helical PriCT‐1 linker domain and the S3H domain are encoded by separate genes (Fig. 6). We also identified one thaumarchaeal CIE, NitAQ6f‐E2, encoding a rolling‐circle replication initiation endonuclease homologous to those of haloarchaeal sphaerolipovirus SNJ1 and several euryarchaeal plasmids (Wang *et al*., 2018b), suggesting that NitAQ6f‐E2 replicates by the rolling‐circle mechanism. Finally, NitGar‐E5 carries an operon consisting of a PolB gene, two copies of a gene encoding a small uncharacterized protein (arCOG08101), and an inactivated RadA homologue (Fig. 6). Similar operons have been previously identified in archaeal genomes and proposed to be involved in DNA repair or regulation of replication (Makarova *et al*., 2014a).

For many CIEs, we could not identify obvious candidates for replication proteins. For instance, the smallest identified CIE, NitUzo‐E2 (2.6 kb), encodes only four predicted proteins, including an integrase, a winged helix‐turn‐helix (wHTH) protein and two hypothetical proteins (Fig. 6). The replication of this element might be initiated by the wHTH protein, as in the case of Reps from the IncP‐1 family plasmids (Konieczny *et al*., 2014). However, given that wHTH proteins also are likely to be involved in transcription regulation, functional assignment without experimental verification appears premature. Overall, the replication modules of CIEs closely resemble those of ICEs, suggesting frequent transitions between the two types of iMGE. As a case in point, NitVie‐E4 encodes a VirB6 homologue but no other recognizable proteins involved in conjugation, suggesting that this element evolved from an ICE ancestor via the loss of the conjugation apparatus which is consistent with the twice‐smaller size of this element (20.2 kb) compared to that of ICE.

#### *Insertion sequences*

**Figure 7:**
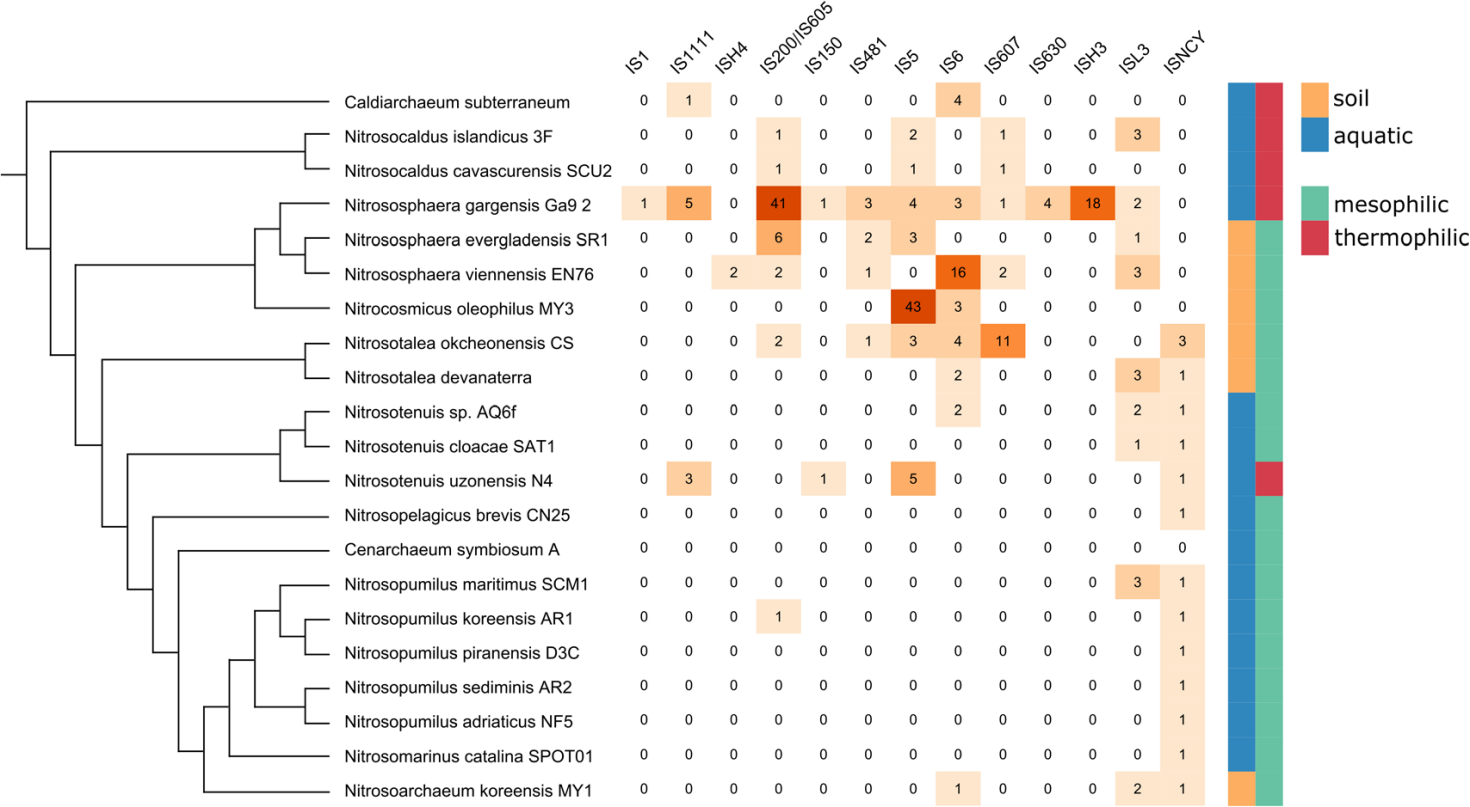
Diversity and distribution of thaumarchaeal insertion sequences. On the left is the schematic cladogram representing the relationships among thaumarchaeal species. The source of isolation is indicated on the right of the figure. The abundance of identified IS elements in each species is shown as a heatmap, with the exact numbers indicated within the corresponding cells. [Color figure can be viewed at wileyonlinelibrary.com]

Although, previous comprehensive analysis of the IS diversity in archaea did not include representatives from the Thaumarchaeota (Filée *et al*., 2007), similar to many other archaea and bacteria, thaumarchaeal genomes are extensively parasitized by IS‐like transposons. We identified 244 IS belonging to 13 families across 20 thaumarchaeal genomes (Fig. 7, Supporting Information Table S1). The majority of thaumarchaeal IS encode transposases of the DDE superfamily (11 IS families), whereas transposases of the HUH and serine recombinase superfamilies are characteristic of the IS*200*/IS*605* and IS*607* families, respectively. Notably, IS*150* family elements have not been previously described in archaea (Filée *et al*., 2007). There is considerable variation in both the copy number and diversity of IS elements among thaumarchaeal species (Fig. 7). Whereas most thaumarchaea carry only a few IS per genome, six species contain ten or more copies of different transposons (Fig. 1C, Supporting Information Table S1). The highest number of IS elements is found in *Ca*. Ns. gargensis Ga9_2 which carries 83 IS from 11 different families, with IS*200*/IS*605* being the dominant one (Fig. 7). There are signs of transposon proliferation and expansion for certain IS families. For instance, IS*5* elements in *Ca*. Nitrocosmicus oleophilus MY3 are found in 43 copies per chromosome, the largest for any thaumarchaeal IS family, whereas in all other species, they are present in low copy numbers or are lacking altogether. Some of the IS families are restricted to a single thaumarchaeal species (IS*1*, IS*4*, IS*630*, IS*H3*; Fig. 7), suggesting a recent horizontal acquisition, but the sources of these transfers remain to be investigated.

### *iMGE‐encoded CRISPR arrays*

**Figure 8:**
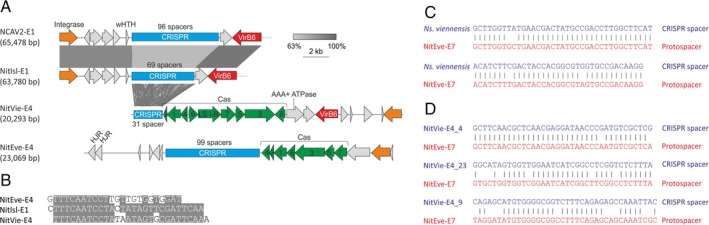
CRISPR arrays carried by thaumarchaeal iMGE. A. Loci of iMGE‐carried stand‐alone CRISPR arrays and CRISPR‐Cas systems. CRISPR arrays are shown as blue rectangles with the number of spacers indicated. *cas* genes are shown in green and indicated with the corresponding numbers. LS, large subunit; HJR, Holliday junction resolvase; wHTH, winged helix‐turn‐helix. B. Alignment of the CRISPR repeat sequences from NitIsl‐E1/NCAV2‐E1, NitVie‐E4 and NitEve‐E4 iMGE. C. Matches between the chromosomal CRISPR spacers (blue) and iMGE (red). D. Matches between the iMGE‐carried CRISPR spacers (blue) and iMGE (red). [Color figure can be viewed at wileyonlinelibrary.com]

Four iMGE, namely, 2 ICE (NCAV2‐E1 and NitIsl‐E1) and 2 CIE (NitVie‐E4 and NitEve‐E4), were found to carry CRISPR arrays (Fig. 8A). In the two CIEs, the CRISPR arrays are adjacent to complete suites of Type‐IB *cas* genes, including apparently functional adaptation and effector modules. By contrast, in the ICEs, the CRISPR arrays are not accompanied by *cas* genes. As aforementioned, NCAV2‐E1 and NitIsl‐E1 are closely related (Fig. 5A), and the major differences between the two ICEs involve the corresponding CRISPR arrays (Fig. 8A). Despite identical repeat sequences, the number of CRISPR spacers is different between the two elements (96 in NCAV2‐E1 versus 69 in NitIsl‐E1). Furthermore, only 43 spacers are shared between NCAV2‐E1 and NitIsl‐E1, whereas the rest of the spacers were apparently divergently acquired following the diversification of the two *Nitrosocaldus* strains, suggesting active exposure to distinct MGEs. For such *in trans* insertion of spacers by the host adaptation machinery to occur, the repeats in the iMGE should be (nearly) identical to those in the host CRISPR array. This is indeed the case, as the repeat sequences of NCAV2‐E1/NitIsl‐E1 are identical to those of the endogenous CRISPR array #3 of *Ca*. Nc. cavascurensis SCU2 which is accompanied by an apparently functional Type I‐B *cas* genes, including the adaptation module (Abby *et al*., 2018). Notably, the repeat sequence of NitVie‐E4 is closely related to that of NCAV2‐E1/NitIsl‐E1 (Fig. 8B), despite the lack of shared spacers and presence of the *cas* genes in NitVie‐E4. Although the repeat sequence of NitEve‐E4 is more divergent, its comparison with the repeat sequences from the other iMGEs (Fig. 8B) indicates that they all might be related.

To gain insight into the provenance of the iMGE‐encoded CRISPR‐Cas systems, we assessed the positions of the corresponding Cas1 proteins, the signature proteins of the CRISPR‐Cas systems, in the global Cas1 phylogeny (Makarova *et al*., 2018). The Cas1 from NitVie‐E4 was nested among bacterial Cas1 homologues from Type I‐B systems, whereas Cas1 from NitEve‐E4 forms a clade with homologues from *Ns. viennensis* EN7 and *Nitrosopumilus* sp. LS, which was nested among Cas1 associated with Type‐III CIRSPR‐Cas systems (Makarova *et al*., 2018). This phylogenetic position suggests that the Type I‐B CRISPR‐Cas systems carried by the two thaumarchaeal iMGE have been independently acquired from distinct sources. Furthermore, the similarity between the repeat sequences of the iMGE‐carried stand‐alone CRISPR arrays and the host array accompanied by *cas* genes suggests that the former evolved from the latter through the loss of the *cas* genes.

To investigate potential interplay between thaumarchaeal iMGE and CRISPR‐Cas systems, we first examined if any of the cellular CRISPR spacers target the identified iMGE. Two spacers in the genome of *Ns. viennensis* EN7 produced significant matches (95% and 94% identity, respectively) to the provirus NitEve‐E7 (Fig. 8C). Notably, both spacers targeted different regions of the gene for the DJR MCP. Next, we analysed if the CRISPR spacers encoded by the four iMGEs target other iMGEs. Three spacers from the NitVie‐E4 were found to match (95% [*E* = 2.5e‐12], 79% [*E* = 1.1e‐05] and 74% [*E* = 1.35e‐04] identity, respectively) the NitEve‐E7 provirus, with one of the spacers (NitVie‐E4_4) targeting the DJR MCP gene (Fig. 8D) at a different region than the two spacers from the bona fide chromosomal *Ns. viennensis* EN7 CRISPR array. The similarities between the NitVie‐E4_23 and NitVie‐E4_9 spacers and their targets are at the boarder of significance. Thus, as a control, BLASTN search (word size 8, identity over full length of spacer > 70% and *E*‐value <0.001) of spacer matches was performed against the *Escherichia coli* genome, which is of a similar size and GC content as our thaumarchaeal iMGE database. No spacer hits with the same thresholds were found in the control search. Furthermore, given that all five spacers (two from the host CRISPR array and three from NitVie‐E4) with identifiable protospacers target the same provirus, it appears likely that these two matches are true positives. Finally, *Ns. viennensis* and *Ns. evergladensis* are both soil‐dwellers (Tourna *et al*., 2011; Zhalnina *et al*., 2014). These observations suggest that the mobile CRISPR loci mediate conflicts between different iMGE competing in the same environment. Obviously, experimental validation is needed to corroborate this conjecture and assess its generality.

### *Functional potential of thaumarchaeal iMGE*

**Figure 9:**
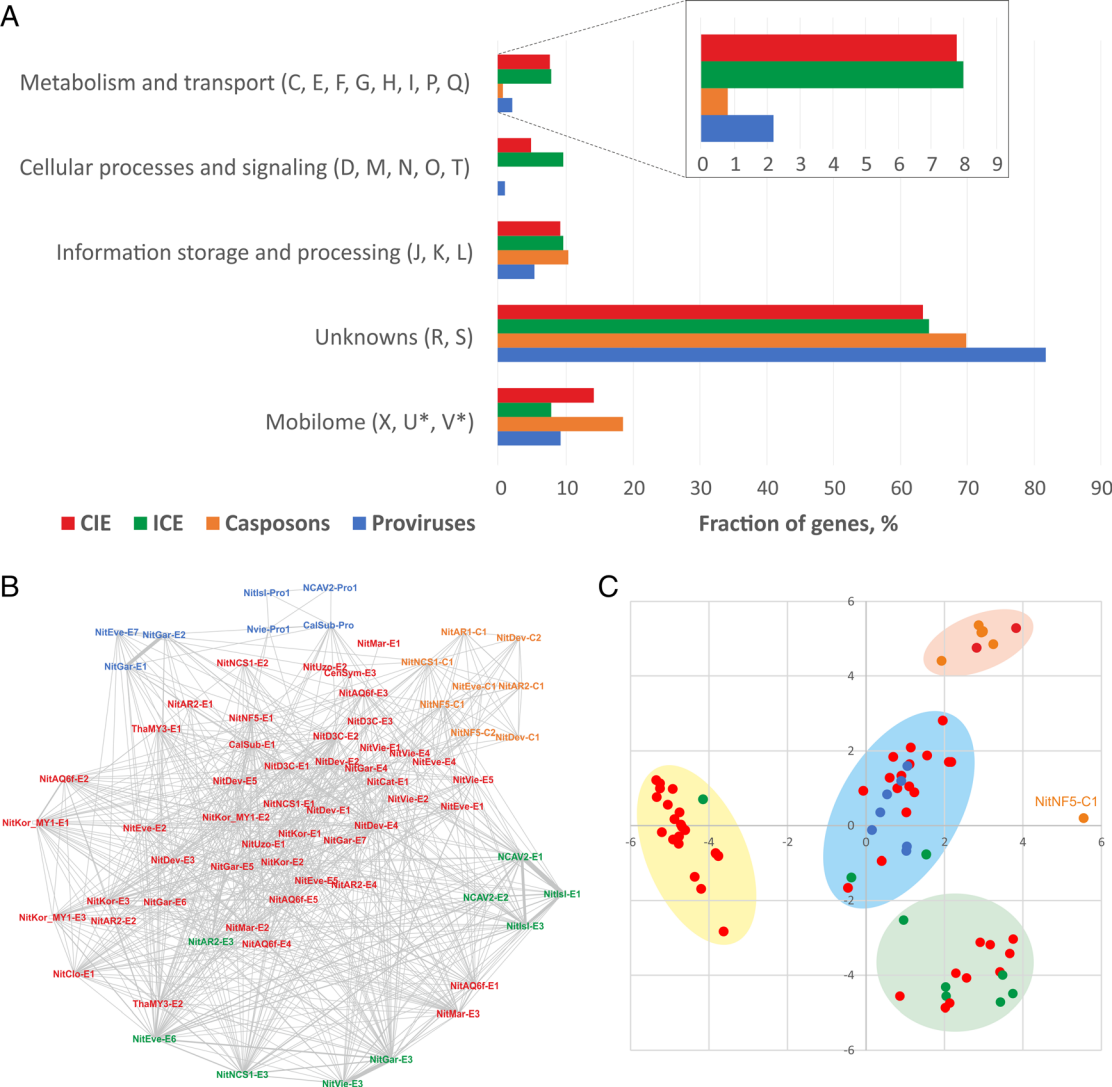
Comparative genomics of thaumarchaeal iMGE. A. Classification of genes from the four classes of iMGE into arCOG functional categories. Note that arCOG categories U (Intracellular trafficking, secretion and vesicular transport) and V (Defence mechanisms) are herein included into the ‘Mobilome’ category. B. Network of thaumarchaeal iMGE based on the shared arCOGs. The nodes correspond to iMGE, whereas the connecting edges represent shared arCOGs. The four iMGE classes are colour‐coded and the key is provided in panel A. C. Classical multidimensional scaling analysis of iMGE. The four iMGE classes are colour‐coded and the key is provided in panel A. [Color figure can be viewed at wileyonlinelibrary.com]

To study the distribution and diversity of functions encoded by different classes of thaumarchaeal iMGE, the 2105 iMGE‐encoded proteins were classified into functional arCOG categories (Makarova *et al*., 2015) (Supporting Information data file 1) and further segregated into five broader group (Fig. 9A). These include

i. ‘Metabolism and transport’ (arCOG categories C, E, F, G, H, I, P and Q);

ii. ‘Cellular processes and signaling’ (arCOG categories D, M, N, O and T);

iii. ‘Information storage and processing’ (arCOG categories J, K and L);

iv. ‘Unknowns’ (arCOG categories R and S, and hypothetical proteins which could not be ascribed to arCOGs);

v. ‘Mobilome’ (arCOG categories X, U and V; note that categories ‘U’: ‘Intracellular trafficking, secretion and vesicular transport’ and ‘V’: ‘Defence mechanisms’ containing the conjugation apparatus and various restriction‐modification systems, respectively, are herein included into the ‘Mobilome’ group).

All 21 functional categories recognized in the arCOG database (Makarova *et al*., 2015) were represented among the iMGE proteins. As is typical of archaeal MGE (Makarova *et al*., 2014c), the majority (63%–82%) of proteins from all four iMGE classes lack functional annotation and fall into the ‘Unknowns’ group, with the highest number of such proteins found in proviruses (Fig. 9A). By contrast, the proteins typical of MGE, such as structural virion proteins, integrases, genome packaging ATPases, transposases and other proteins from the ‘Mobilome’ category, represented a core of less than 20% (less than 10% for proviruses and ICE) of the total protein content in each iMGE class. Notably, proviruses and casposons were relatively depleted in proteins of the groups ‘Information storage and processing’ and ‘Cellular processes and signaling’, whereas ICE and CIE carry greater numbers of the so‐called auxiliary metabolic genes (AMG) involved in metabolism and transport compared to proviruses and casposons (Fig. 9A, inset). For instance, many elements encode multicopper oxidases, which have been suggested to assist in the process of ammonia oxidation by producing NO (Schleper and Nicol, 2010; Kozlowski *et al*., 2016). In addition, one element, NitEve‐E6, encodes an ammonia monooxygenase subunit C (AmoC; hit to PFAM profile PF04896.12, HHpred probability = 100%) and two iMGE encode nitrogen regulatory protein PII (HHpred probabilities > 99%), and might actively participate in nitrogen cycling in soil environments, as has been recently proposed for putative AmoC‐encoding marine thaumarchaeal viruses assembled from metagenomic data (Ahlgren *et al*., 2019). In addition, iMGE were found to encode various dehydrogenases, stress response proteins, different membrane transporters of cations and drugs, chemotaxis protein receiver domains and many more (Supporting Information data file 1). The discovery of this diverse protein repertoire suggests that conjugative and cryptic elements play important roles in host adaptation and affect the fitness and survival of their hosts.

### *All thaumarchaeal iMGE are connected in a gene sharing network*

Comparison of the gene (arCOG) content across the four classes of iMGE shows that all elements are connected to each other within a gene sharing network (Fig. 9B), indicating that some iMGE carry genes with broad distribution across different iMGE classes. Nevertheless, the two subgroups of proviruses (*Caudovirales* and DJR MCP‐encoding proviruses, respectively) and casposons formed discernible clusters within this network, suggesting that, in the case of iMGE with relatively small genomes, a small set of core genes is sufficient to hold the (sub)classes together. By contrast, CIE and ICE were largely intermixed. Embedding the iMGE distance matrix into a 2‐D space using Classical Multidimensional Scaling (CMDS) analysis (Borg and Groenen, 2005), revealed four clusters of elements (Fig. 9C). However, these clusters were not homogeneous with respect to the four iMGE classes. For instance, CIEs were distributed across all four clusters, whereas ICEs were present in three clusters. Notably, NitNF5‐C1, the largest of the identified casposons (Fig. 4A), did not cluster with other casposons but was an outlier (Fig. 9C). This is not surprising, given that this casposon, besides the casposon‐specific proteins, encodes several other proteins, including XerC‐like tyrosine recombinase, that are shared with many other iMGE.

Analysis of the iMGE gene content revealed several protein families broadly distributed in iMGE (Table 1) which provide connectivity within the network. These include not only the XerC/XerD and Cas1 family integrases which, primarily, the former family, are essential for mobility and, thus, carried by the vast majority of iMGE, but also different families of transcription regulators, components of restriction modification and conjugation systems and several protein families potentially contributing to the host fitness and adaptation. For instance, 16 iMGE encode universal stress response proteins of the UspA family (Table 1). The proteins of the UspA family have been shown to play regulatory and protective roles to enable microbial adaptation and survival under various environmental stresses, such as nutrient starvation, drought, extreme temperatures, high salinity, the presence of antibiotics and heavy metals and other forms of stress (Vollmer and Bark, 2018). The connectivity of the iMGE network and the extent of gene sharing suggest that the thaumarchaeal mobilome has been shaped by three major processes, namely, (1) horizontal gene exchange, (2) independent acquisition of homologous genes from the host and (3) evolutionary transitions between different iMGE classes, in particular, between the CIE and ICE.

**Table 1:** Top 20 most common arCOGs from the thaumarchaeal iMGE.

Count	arCOG	Category	Annotation
33	arCOG01245	X	XerD/XerC family integrase
17	arCOG01242	X	XerD/XerC family integrase
16	arCOG02053	T	UspA family nucleotide‐binding protein
13	arCOG00606	R	CBS domain
12	arCOG08677	S	Zn‐ribbon domain containing protein
11	arCOG02626	V	Type I restriction‐modification system, S subunit
10	arCOG01452	V	CRISPR‐associated protein Cas1
9	arCOG08805	V	CopG/RHH family DNA binding protein
9	arCOG03914	Q	Multicopper oxidase
9	arCOG00602	R	CBS domain containing protein
9	arCOG00608	K	Predicted transcriptional regulator with C‐terminal CBS domains
9	arCOG02632	V	Type I restriction‐modification system, methyltransferase subunit
8	arCOG01471	R	Hemerythrin HHE cation binding domain containing protein
8	arCOG01981	K	Transcription initiation factor TFIIB
7	arCOG15271	X	Casposon associated protein‐primed PolB family polymerase
7	arCOG04559	P	Membrane transporter of cations and cationic drugs
7	arCOG02868	O	Protein‐disulfide isomerase
7	arCOG07844	S	VirB6/TrbL; membrane protein associated with conjugation system
6	arCOG14992	S	Uncharacterized protein conserved in casposons
6	arCOG00878	V	Type I restriction‐modification system, restriction subunit

## Discussion

Based on functional considerations and mode of propagation, thaumarchaeal iMGE can be categorized into five classes, namely, proviruses, casposons, ICE, CIE and the short IS‐like transposons. Whereas IS‐like transposons generally consist of 1 or 2 genes, those of the other four classes encompass multiple genes and display great diversity in terms of genomic complexity and functional content. All five classes of iMGE found in thaumarchaea are also present in other archaea (e.g. phylum Euryarchaeota) and bacteria although some of the classes have not been thus far identified in certain archaeal and bacterial lineages. For instance, casposons and viruses of the order *Caudovirales* have not been detected in members of the phylum Crenarchaeota. This might be due to insufficient sampling or to genuine lack of these elements in this archaeal phylum. By contrast, bacteria are known to contain additional classes of iMGE that have not been detected in archaea, including thaumarchaea. These include composite DNA transposons which, in addition to the transposase genes, carry diverse passenger genes, such as those for antibiotic resistance (Nicolas *et al*., 2015); various pathogenicity islands and phage‐inducible chromosomal islands that are induced upon phage infection and hijack the virus particle for intercellular transmission (Novick and Ram, 2016; 2017); mobile integrons, complex genetic platforms that allow bacteria to evolve rapidly through the acquisition, excision and shuffling of genes found in mobile elements known as cassettes (Escudero *et al*., 2015); or pipolins, a recently characterized group of bacterial iMGE encoding primer‐independent DNA polymerases (Redrejo‐Rodríguez *et al*., 2017). However, given our limited understanding on the archaeal mobilome and especially the diversity of iMGE, it cannot be ruled out that counterparts to some of these bacterial iMGE classes in thaumarchaea are awaiting discovery. The CIE class is particularly enigmatic and might include functionally distinct classes of iMGE.

In addition to proviruses related to tailed viruses of the order *Caudovirales*, which have been previously observed in thaumarchaeal genomes and also detected by several metagenomics studies (Chow *et al*., 2015; Labonté *et al*., 2015; Ahlgren *et al*., 2019; López‐Pérez *et al*., 2018), we identified proviruses encoding the DJR MCP, one of the most widely distributed and diverse groups of dsDNA viruses in all three domains of life (Krupovic and Bamford, 2008a; Krupovic and Koonin, 2015; Yutin *et al*., 2018). Although the number of identified archaeal viruses with the DJR MCP is small, phylogenetic analysis suggests a coevolution of this virus group with the major archaeal lineages, including Thaumarchaeota. If validated by broader studies, this conclusion would parallel the apparently ancient evolutionary association of the *Caudovirales* with thaumarchaea (Krupovic *et al*., 2011). Thus, at least these two groups of viruses can be confidently traced to the last common ancestor of the archaea and, in all likelihood, to the last universal cellular ancestor. We did not identify any iMGE related to the archaea‐specific virus groups associated with other archaeal phyla, and whether any of these extend to Thaumarchaeota, remain to be determined. Potentially, some or even many of the CIE, which comprise the majority of the identified thaumarchaeal iMGE (65%), represent novel families of archaeal viruses and plasmids. Systematic experimental induction of the replication of CIE and ICE could be a rewarding exercise, not only from a fundamental standpoint, but also to develop replicons that might serve as much‐needed genetic tools in thaumarchaea. Identification of iMGE in thaumarchaea from diverse environments provides a broad choice of potential replicons that potentially could be tailored for different model organisms. Given their circular topology, CIE and ICE elements with smaller genome sizes (3–12 kbp) appear to be best suited for the development of shuttle vectors for facile genetic manipulation in *Escherichia coli*.

Gene content analysis revealed an extensive pan‐genome of thaumarchaeal iMGE. The MGE‐specific genes, such as those encoding capsid proteins, viral genome packaging ATPases, conjugation proteins, integrases and so forth, constitute but a small fraction of their gene complements (10%–20% of genes). The vast majority of the iMGE genes encode proteins of unknown function. Nevertheless, a substantial fraction of genes represents auxiliary metabolic genes and stress response genes which are likely to play important roles in the adaption of their hosts to new environments, coping with stressful conditions and boosting their metabolic potential. For instance, multicopper oxidases, AmoC and nitrogen regulatory protein PII encoded by iMGE might modulate nitrogen metabolism, whereas UspA family proteins could boost the adaptation and survival of the host cells under various environmental stress conditions. The identification of functionally diverse metabolic and signalling genes in the thaumarcaheal iMGE parallels observations on the gene repertoires of some of the tailed bacterial viruses (Anantharaman *et al*., 2014; Hurwitz and U'Ren, 2016; Roux *et al*., 2016; Roitman *et al*., 2018), in particular, cyanophages that carry photosystem genes and substantially contribute to the host metabolism (Sharon *et al*., 2009; Thompson *et al*., 2011; Fridman *et al*., 2017). Taken together, these observations indicate that, at least, in the case of iMGEs with larger genomes, these elements should be considered more as symbionts of their hosts than simple genomic parasites or ‘junk DNA’.

Although metabolism‐related genes appear to be more prevalent in CIE and ICE, all four classes of iMGE share a substantial fraction of genes. Accordingly, the evolutionary relationships between these iMGE are most adequately represented as a gene‐sharing network similar to those that have been previously constructed for double‐stranded DNA viruses (Jachiet *et al*., 2014; Iranzo *et al*., 2016a,b; Bolduc *et al*., 2017). The extensive gene sharing can be explained by three nonmutually exclusive scenarios, including (1) horizontal gene exchange, (2) independent acquisition of homologous genes from various sources and (3) evolutionary transitions between different iMGE classes. Gene content similarity suggests that such transitions indeed occurred on multiple occasions between CIE and ICE, and involved the loss/acquisition of the genes encoding the conjugative apparatus.

The vast majority of known CRSIPR‐Cas systems are encoded by cellular organisms and deployed to counter the replication of MGE, but some MGE also carry functional CRISPR‐Cas systems. For instance, CRISPR‐Cas systems and stand‐alone CRISPR arrays have been identified in a number of prophages (Hargreaves *et al*., 2014; Chénard *et al*., 2016; Zheng *et al*., 2016; Garneau *et al*., 2018) and in the case of a *Vibrio*‐infecting bacteriophage have been shown to target for destruction a pathogenicity island integrated in the host genome (Seed *et al*., 2013). By contrast, a subgroup of Tn7‐like transposons has been hypothesized to employ the encoded CRISPR‐Cas system for CRISPR‐guided transposition (Peters *et al*., 2017). We identified four iMGE carrying CRISPR arrays, which in two cases were accompanied by complete suites of *cas* genes. The majority of spacers did not match any known viruses, mostly likely, due to the current lack of data on the thaumarchaeal mobilome. Interestingly, however, several spacers carried by a CIE matched one of the proviruses, apparently, indicative of an antagonistic interaction between iMGE residing in the same habitat. Consequently, the CRISPR‐carrying CIE and the host cell appear to coexist in a symbiotic relationship, whereby the CIE provides a protection against a presumably more harmful provirus. Identification of the CRISPR loci in MGE described here and elsewhere are consistent with the ‘guns‐for‐hire’ concept whereby MGE capture and repurpose various host defence systems (Koonin and Krupovic, 2015). Collectively, our results provide insights into the diversity and evolution of the thaumarchaeal mobilome and illuminate its potential impact on the functioning and adaptation of the host cells.

## Experimental procedures

### *Identification of iMGE*

Complete or near‐complete thaumarchaeal genomes were downloaded from the NCBI database. We employed three different strategies to search for the iMGEs. (i) The genomes were analysed for the presence of gene clusters, previously denoted as ‘dark matter’ islands, enriched in ORFans and uncharacterized genes with a very narrow phyletic distribution (Makarova *et al*., 2014c). (ii) The second approach was based on identification of genes encoding signature proteins typical of different archaeal MGE groups. These included major capsid and genome packaging proteins representing different families of archaeal viruses, protein‐primed family B DNA polymerases, rolling‐circle replication initiation endonucleases and SSV‐type DnaA‐like AAA+ ATPase. Whenever a homologue of the signature MGE gene was identified in the cellular genome, the search was repeated with the identified thaumarchaeal homologue and its genomic context was analysed for the presence of additional MGE‐derived genes using blastp. (iii) The third strategy involved systematic genome context analysis of genes encoding for integrases of the tyrosine recombinase superfamily. The searches were performed against the dataset of thaumarchaeal genomes using tblastn and integrase sequences from each newly identified thaumarchaeal iMGE as queries. The three approaches produced overlapping, yet complimentary results. In the next step, the potential iMGEs were analysed for the presence of signatures of site‐specific recombination.

### *Identification of insertion sequences*

IS elements were predicted and classified into families using the ISsaga platform (Varani *et al*., 2011). The ‘probable false‐positive’ predicted by ISsaga were excluded from the final results. Exact coordinates for all identified IS elements are provided in Supporting Information Table S3.

### *Determination of the integration sites*

The precise boundaries of integration were defined based on the presence of direct repeats corresponding to attachment sites or target site duplications. The direct and inverted repeats were searched for using Unipro UGENE (Okonechnikov *et al*., 2012). Whenever possible, additional validation of the MGE integration sites was obtained by comparing sequences of genomes containing the putative iMGEs with those of closely related genomes that do not contain such insertions using blastn algorithm.

### *Annotation of the iMGE genes*

For each analysed gene, the functional annotations were assigned using the PSI‐BLAST program with position specific scoring matrixes derived from arCOG alignments (Altschul *et al*., 1997). To detect remote homology, additional searches were performed using PSI‐BLAST (Altschul *et al*., 1997) against the nonredundant protein database at NCBI and HHpred against the PDB, CDD, SCOPe and Pfam databases available through the MPI Bioinformatics Toolkit (Zimmermann *et al*., 2018).

### *Network analysis*

The number of distinct arCOGs shared between a pair of elements (*S*_*ij*_) was counted in annotated iMGEs. In the network representation the thickness of the line, connecting two iMGE is proportional to *S*_*ij*_. The distance between two elements with the respective numbers of genes *X*_*i*_ and *X*_*j*_ is calculated as ‐ln(*S*_*ij*_/sqrt(*X*_*i*_ *X*_*j*_)). The iMGE distance matrix was embedded into a 2‐D space using the classical multidimensional scaling (*cmdscale* function in R).

### *Phylogenetic analysis*

For phylogenetic analysis, MCP and ATPase sequences from each (pro)virus were concatenated and aligned using MUSCLE (Edgar, 2004). Poorly aligned (low information content) positions were removed using the Gappyout function of Trimal (Capella‐Gutierrez *et al*., 2009). The final alignment contained 462 positions. The maximum likelihood phylogenetic tree was constructed using the PhyML program (Guindon *et al*., 2010) with the automatic selection of the best‐fit substitution model for a given alignment. The best model identified by PhyML was RtREV +G6 + I + F. The tree was rooted with sequences of bacterial tectiviruses. The branch support was assessed using aBayes implemented in PhyML.

### *Genome comparisons*

The genomes of iMGE were compared and visualized using EasyFig v2.1 with tblastx algorithm (Sullivan *et al*., 2011). The complete genomes of closely related *Nitrosocaldus* strains, *Ca*. Nc. cavascurensis SCU2 and *Ca*. Nc. islandicus 3F were compared using progressiveMauve with default parameters (Darling *et al*., 2010).

## Supporting information


**Appendix S1.** Supporting information

**Table S1.** Supporting information

**Table S2.** Supporting information

**Table S3.** Supporting information
